# Investigating the robustness of the classical enzyme kinetic equations in small intracellular compartments

**DOI:** 10.1186/1752-0509-3-101

**Published:** 2009-10-08

**Authors:** Ramon Grima

**Affiliations:** 1 School of Biological Sciences, Centre for Systems Biology at Edinburgh, University of Edinburgh, UK

## Abstract

**Background:**

Classical descriptions of enzyme kinetics ignore the physical nature of the intracellular environment. Main implicit assumptions behind such approaches are that reactions occur in compartment volumes which are large enough so that molecular discreteness can be ignored and that molecular transport occurs via diffusion. Though these conditions are frequently met in laboratory conditions, they are not characteristic of the intracellular environment, which is compartmentalized at the micron and submicron scales and in which active means of transport play a significant role.

**Results:**

Starting from a master equation description of enzyme reaction kinetics and assuming metabolic steady-state conditions, we derive novel mesoscopic rate equations which take into account (i) the intrinsic molecular noise due to the low copy number of molecules in intracellular compartments (ii) the physical nature of the substrate transport process, i.e. diffusion or vesicle-mediated transport. These equations replace the conventional macroscopic and deterministic equations in the context of intracellular kinetics. The latter are recovered in the limit of infinite compartment volumes. We find that deviations from the predictions of classical kinetics are pronounced (hundreds of percent in the estimate for the reaction velocity) for enzyme reactions occurring in compartments which are smaller than approximately 200 nm, for the case of substrate transport to the compartment being mediated principally by vesicle or granule transport and in the presence of competitive enzyme inhibitors.

**Conclusion:**

The derived mesoscopic rate equations describe subcellular enzyme reaction kinetics, taking into account, for the first time, the simultaneous influence of both intrinsic noise and the mode of transport. They clearly show the range of applicability of the conventional deterministic equation models, namely intracellular conditions compatible with diffusive transport and simple enzyme mechanisms in several hundred nanometre-sized compartments. An active transport mechanism coupled with large intrinsic noise in enzyme concentrations is shown to lead to huge deviations from the predictions of deterministic models. This has implications for the common approach of modeling large intracellular reaction networks using ordinary differential equations and also for the calculation of the effective dosage of competitive inhibitor drugs.

## Background

The inside of a cell is a highly complex environment. In the past two decades, detailed measurements of the chemical and biophysical properties of the cytoplasm have established that the conditions in which intracellular reactions occur are, by and large, very different than those typically maintained in laboratory conditions. One of the outstanding differences between *in vivo *and *in vitro *conditions, is that in the former, biochemical reactions typically occur in minuscule reaction volumes [1]. For example, in eukaryotic cells, many biochemical pathways are sequestered within membrane-bound compartments, ranging from ~50 nm diameter vesicles to the nucleus, which can be several microns in size [2]. It is also found that the total concentration of macromolecules inside both prokaryotic and eukaryotic cells is very large [3,4], of the order of 50 - 400 mg/ml which implies that between 5% and 40% of the total intracellular volume is physically occupied by these molecules [5]. The concentration of these crowding molecules is highly heterogeneous (see for example [6]), meaning that typically one will find small pockets of intracellular space, characterized by low macromolecular crowding, surrounded by a "sea" of high crowding; such pockets of space may serve as effective compartments where reactions may occur more easily than in the rest of the cytosol. Analysis of experimental data for the dependence of diffusion coefficients with molecular size suggests the length scale of such effective compartments is in the range 35-50 nm [7], a size comparable to that of the smallest vesicles. The significant crowding also suggests that frequently an active means of transport such as vesicle-mediated transport, may be more desirable than simple diffusion as a means of intracellular transport.

The volume of a spherical cavity of space of diameter 50 nm is merely ~6.5 × 10^-20 ^liters, an extremely small number compared to the typical macroscopic reaction volumes of *in vitro *experiments (experimental attolitre biochemistry is still in its infancy - see for example [8]). These very small reaction volumes imply that at physiologically relevant concentrations (nano to millimolar), the copy number of a significant number of intracellular molecules is very small [1] and consequently that intrinsic noise cannot be ignored; for example 255 *μM *corresponds to an average of just 10 molecules in a 50 nm vesicle and fluctuations about this mean of the order of 3 molecules [9].

The traditional mathematical framework of physical chemistry ignores the basic physical properties of the intracellular environment. Kinetics are described by a set of coupled ordinary differential equations which implicitly assume (i) that the reaction compartment is so large that molecular discreteness can be ignored and that hence integer numbers of molecules per unit volume can be replaced by a continuous variable, the molar concentration. Since the number of molecules is assumed to be very large, stochastic fluctuations are deemed negligible and the equations are hence deterministic; (ii) the reaction compartment is well-stirred so that homogeneous conditions prevail throughout [9]. Both assumptions can be justified for reactions occurring in a constantly stirred reactor of macroscopic dimensions. However if diffusion is the dominant transport process inside the compartment then the homogeneity assumption holds only if the volume is small enough so that in the time between successive reactions, a molecule will diffuse a distance much larger than the size of the compartment. This comes at the expense of the first assumption. It hence appears natural that for intracellular applications, the first assumption, namely that of deterministic kinetics cannot be justified *a priori*. The second assumption can be justified if reactions are localized in sufficiently small parts of the cell and in particular for reaction-limited processes i.e. those for which the typical time for two molecules to meet each other via diffusion is much less than the typical time for them to react if they are in close proximity. For such conditions, a molecule will come within reaction range several times before participating in a successful reaction, in the process sampling the compartment many times which naturally leads to well-mixed conditions [9-11].

**Figure 1:**
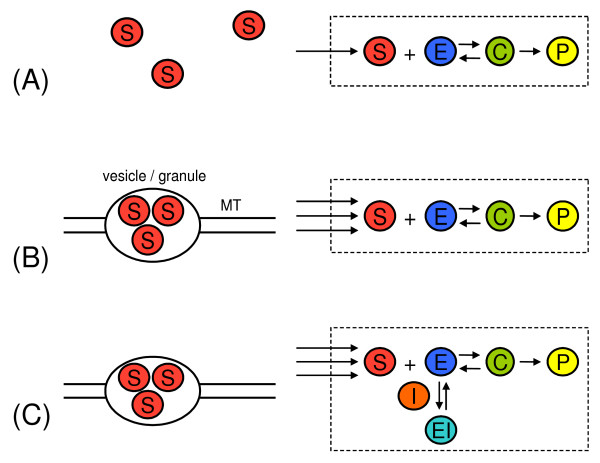
**Schematic illustrating the three models considered in this article**. (A) Model I: Michaelis-Menten reaction occurring in a compartment volume of sub-micron dimensions (shown by dashed rectangle). Substrate input into compartment occurs via a Poisson process i.e. diffusion-mediated substrate transport. (B) Model II: As for Model I but now substrate is input into compartment in groups or bursts of *M *molecules at a time i.e. vesicle-mediated substrate transport along microtubules (MT). (C) Model III: Michaelis-Menten reaction with competitive inhibitor (*I*) occurring in a small subcellular compartment. Substrate transport as in previous two models.

In this article we seek to understand the magnitude of deviations from the classical kinetic equations in small intracellular compartment volumes. We specifically focus on the case of reaction-limited enzyme reactions which allows us to relax the first assumption of physical chemistry while keeping the second one; this makes the mathematics tractable. We quantify deviations from classical kinetics in the context of the Michaelis-Menten (MM) equation; this is the cornerstone of present day enzyme kinetics and is a derivative of the traditional deterministic mathematical framework based on ordinary differential equations. In steady-state metabolic conditions, it is predicted to be exact. Thus this equation is ideal as a means to accurately test the effects of small-scale compartmentation on chemical kinetics. We consider three successive biological models of intracellular enzyme kinetics, each one building on the biological detail and realism from the previous one (Figure 1). The models incorporate the intrinsic noisiness of kinetics in small compartments, the details of the substrate transport process to the compartment (diffusion or active transport) and the presence of intra-compartmental molecules other than substrate molecules which may modulate the enzyme-catalyzed process e.g. inhibitors. On the macroscopic level, i.e. for large volumes, the steady-state kinetics of all models conform to the MM equation. We test whether this equation holds on the on the length scale of small intracellular compartments by deriving the dependence of the ensemble averaged rate of product formation on the ensemble-averaged substrate concentration from the corresponding stochastic models in the steady-state. It is shown via both calculation and stochastic simulation that at these small length scales the MM equation breaks down, being replaced by a new more general equation. Practical consequences of this breakdown are illustrated in the context of the calculation of the effective dosage of enzyme inhibitor drug needed to suppress intra-compartmental enzyme activity by a given amount. To make our approach accessible to readers not familiar with stochastic equations and their analysis, in the Results/Discussion sections we mainly focus on the biological/biophysical context and implications of the models together with the main mathematical results which are verified by simulation. Detailed mathematical derivations and the methods of simulation are relegated to the Methods section.

## Results

### Model I: Michaelis-Menten reaction occurring in a compartment volume of sub-micron dimensions. Substrate input into compartment occurs via a Poisson process

This is the simplest, biologically-relevant case (Figure 1(A)). The reaction scheme is . Substrate molecules (*S*) are continuously supplied inside the compartment at some rate *k*_*in*_, they reversibly bind to enzyme molecules (*E*) with rate constants *k*_0 _(forward reaction) and *k*_1 _(backward reaction) to form transitory enzyme-substrate complex molecules (*C*) which then decay with rate *k*_2 _into enzyme and product molecules (*P*). The substrate input is assumed to be governed by a Poisson process with mean *k*_*in*_; this is consistent with substrate transport to the compartment being dominated by normal diffusion. The enzyme acts as a catalyst, effectively speeding up the reaction by orders of magnitude. It is assumed that diffusion inside the compartment is normal and not rate-limiting on the catalytic process i.e. well-mixed conditions or rate-limited kinetics inside the compartment. Given these conditions we ask ourselves what is the relationship between the reaction velocity and the substrate concentration inside the compartment. The simplest approach consists of writing down the rate equations of traditional physical chemistry:(1)

By imposing steady-state conditions we get the sought-after relationship which is simply the well-known MM equation:(5)

where *K*_*M *_= (*k*_1 _+ *k*_2_)/*k*_0 _is the MM constant, *v*_*max *_= *k*_2 _[*E*_*T*_] is the maximum reaction velocity and square brackets indicate the macroscopic concentrations. We note that steady-state conditions for substrate necessarily require that *k*_*in *_≤ *v*_*max *_otherwise the substrate will continuously accumulate with time. Though this approach is simple and straightforward, as mentioned in the introduction, the assumptions behind the formulation of the rate equations are not consistent with the known physical properties of the cytoplasm. In particular it is clear that if the volume of our compartment is very small (as is the case), the numbers of particles is also quite small, meaning that the concept of a continuous variable such as the average macroscopic concentration has little meaning. Rather we require a mathematical description in terms of discrete, integer numbers of particles and which is stochastic. The natural description of such cases is a master equation which is a differential equation in the joint probability function *π *describing the system [12-15]:(6)

where *π *= *π*(*n*_*S*_, *n*_*C*_, *n*_*P*_), *n*_*Y *_is the integer number of molecules of type Y, Ω is the compartment volume, and  are step operators defined in the Methods section. This equation cannot be solved exactly. However it can be solved perturbatively using the system-size expansion due to van Kampen [12]. This expansion is one in powers of the inverse square root of the compartment volume. In the Methods section, we calculate the first three terms of the expansion, namely those proportional to Ω^1/2^, Ω^0^and Ω^-1/2^. The first term, being the dominant one for large volumes, gives back as expected, the rate equations Eqs. (1)-(4). The second term gives the magnitude of stochastic fluctuations about the macroscopic concentrations. Corrections to the rate equations and to the MM equation (due to small compartment volumes or equivalently due to intrinsic noise) are found by considering the third term. In the rest of the article, instead of using the reaction velocity *v*, we use the normalized reaction velocity, *α*, which is simply the velocity of the reaction, *v*, divided by the maximum reaction velocity, *v*_*max*_. Given some measured intracompartmental substrate concentration, [S*] = ⟨*n*_*S*_/Ω⟩ (angled brackets imply average), the relationship between the normalized reaction velocity predicted by the MM equation (*α*_*M *_= [S*]/(*K*_*M *_+ [S*])) and the actual normalized reaction velocity (*α*), as predicted by our theory, is given by:(7)

where,(8)

Hence the prediction of the MM equation is only correct, i.e. *α *= *α*_*M*_, in the limit of infinitely large compartment volumes, in which case the second term on the left hand side of Eq. (7) will become vanishingly small and can be neglected. For finite compartment volumes, the MM equation is not exact (except in the two limiting cases of *α*_*M *_→ 0 and *α*_*M *_→ 1) but is at best an approximation, even though steady-state conditions are imposed; this is at odds with the prediction of the conventional deterministic theory. An inspection of Eqs. (7) and (8) shows that the magnitude of the deviations from the MM equation depends on the two non-dimensional quantities: (i) *K*_*M*_Ω, a measure of the rate at which enzyme-substrate combination events occur relative to the rate of decay of complex molecules; (ii) [*E*_*T*_]Ω, the total integer number of enzyme molecules in the compartment.

As shown in the Methods section, the MM equation is found to implicity assume that the noise about the macroscopic substrate and enzyme concentrations is uncorrelated (this assumption has generally been found to be at the heart of many macroscopic models - for example see [16]); properly taking into account these non-zero correlations leads to the corrections encapsulated by Eqs. (7) and (8). These correlations are expected to be small in two particular cases: (i) if *K*_*M *_is large; in this case when substrate molecules combine with an enzyme to form a complex, the latter dissociates very quickly back into free enzyme and thus successive enzyme-substrate events to the same enzyme molecule are bound to be almost independent of each other. The opposite situation of small *K*_*M *_would imply that the bottleneck in the catalytic process is the decay of complex rather than enzyme-substrate combination; if a successful combination occurs, the next substrate to arrive to the same enzyme molecule would have to wait until the complex decays, naturally leading to correlations between successive enzyme-substrate combination events. (ii) if the total number of enzyme molecules is large; in such a case, at any one time, the noise about the macroscopic concentrations will be the sum total from a large number of enzymes, each at a different stage in the catalytic process and each independent from all others, which naturally dilutes any temporal correlations.

**Figure 2:**
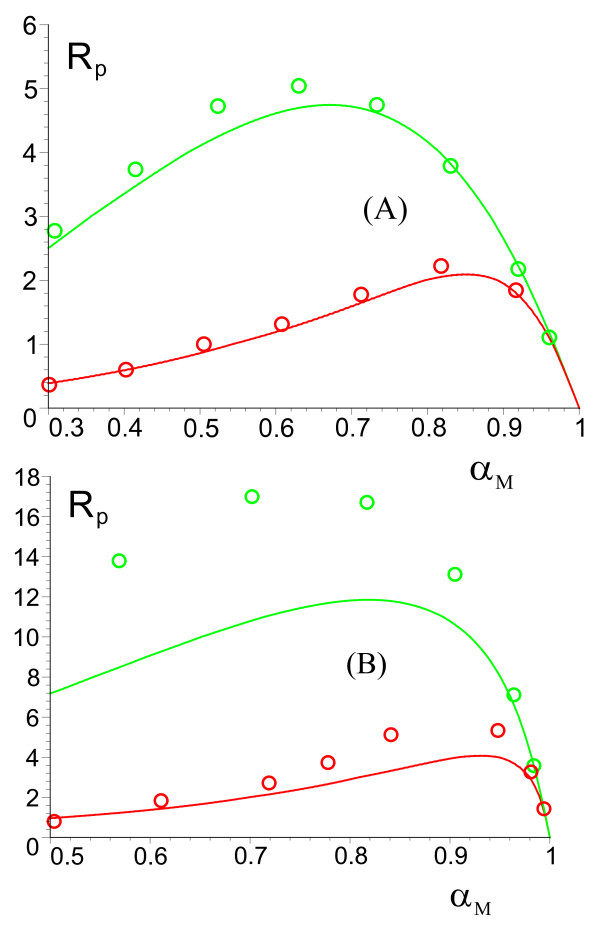
**Deviations from the predictions of the MM equation for diffusion-mediated substrate transport**. (Model I) Plot of the Percentage Error in reaction velocity, *R*_*p *_= 100|1 -*α*_*M*_/*α*|, versus the normalized reaction velocity of the MM equation, *α*_*M *_for 10 enzymes (green) and 100 enzymes (red) with *K*_*M *_= 10 *μM *in compartments with diameter 100 nm (A) and 50 nm (B). The solid lines show the theoretical predictions, as encapsulated by Eqs. (7) and (8); the data points are obtained by stochastic simulation (see Methods for details).

To estimate the magnitude of the deviations from the MM equation inside cells, we use the above two equations, Eqs. (7) and (8), to compute the absolute percentage error *R*_*p *_= 100|1 - *α*_*M*_/*α*|. These estimates are also computed by stochastic simulation of the master Eq. (6), using the exact stochastic simulation algorithm of Gillespie [10] (see Methods for details regarding the method of simulation); this provides a direct test of the theory. Figure 2 shows the typical dependence of *R*_*p *_on *α*_*M*_, as predicted by both theory (solid lines) and simulation (data points). Generally the agreement between the two is found to be very good; discrepancies increase as *K*_*M *_and compartment volume decrease but are small for parameter values realistic for intracellular conditions. The maxima of such plots gives the maximum absolute percentage error which is a measure of the maximum expected deviations from the MM equation. Table 1 summarizes these estimates (theory and simulation) over wide ranges of the parameters typical of *in vivo *conditions: *K*_*M *_= 10 *μM *- 1000 *μM *[17], enzyme copy numbers of ten and one hundred per compartment which correspond to enzyme concentrations ranging from 4 *μM *to 2.5 mM and compartment diameters ranging from 50 nm to 200 nm. Note that the maximum deviations from the MM equation are estimated to be less than approximately 20% and typically just a few percent over large ranges of parameter values - this robustness of the MM equation with respect to intrinsic molecular noise is indeed surprising, since strictly speaking it is only valid for infinite compartment volumes.

**Table 1:** Maximum Percentage error in reaction velocity from prediction of the MM equation for Model I.

D/nm	*K*_*M *_= 10 *μM*	100 *μM*	1000 *μM*	Copy No.
50	*11.83 ***[17.00]**	*4.09 ***[4.33]**	*0.59*	10
100	*4.74 ***[5.00]**	*0.73 ***[0.74]**	*0.08*	10
200	*0.90*	*0.10*	*0.01*	10

50	*3.98 ***[5.33]**	*1.88 ***[2.02]**	*0.43*	100
100	*2.10 ***[2.23]**	*0.52 ***[0.52]**	*0.07*	100
200	*0.61*	*0.09*	*0.01*	100

The copy number indicates the total number of enzyme molecules per compartment. Values in bold and in square brackets are those estimated by simulation; the italic values are obtained from the derived theoretical expressions, Eqs. (7) and (8).

The theory is always found to underestimate the actual deviations predicted by simulations; hence the theoretical expressions provide a quick, convenient way by which one can generally estimate a lower bound on the deviations to be expected from the MM equation without the need to perform extensive stochastic simulation.

### Model II: Michaelis-Menten reaction occurring in a compartment volume of sub-micron dimensions. Substrate is input into compartment in groups or bursts of M molecules at a time

Model I captures the basics of a general enzyme-catalyzed process occurring in a small intracellular compartment. In this section we build upon this model to incorporate further biological realism. In particular, in the previous model we assumed that substrate input can be well described by a Poisson process, where one molecule at a time is fed into the compartment with some average rate *k*_*in*_. This is the simplest possible assumption and approximates well the situation in which molecules are brought to the compartment via normal diffusion. However there are many situations where this may not be the case; we now describe two such cases.

The intracellular condition of macromolecular crowding limits the Brownian motion of molecules in the cytoplasm, this being reflected in the relatively small diffusion coefficients measured *in vivo *compared to those known *in vitro *for moderately to relatively large molecules. Experiments with inert tracer particles in the cytoplasm of Swiss 3T3 cells show that the *in vivo *diffusion coefficient is an order of magnitude less than that *in vitro *for molecules with hydrodynamic radius 14 nm and diffusion becomes negligibly small for molecules larger than approximately 25 nm [7]; similar results have been obtained in Xenopus neurons [18] and skeletal muscle myotubes [19]. If diffusion is considerably hindered, one expects active transport to become a more desirable mode of transport. Indeed there exists ample evidence for the active transport of macromolecules: they are typically packaged in a vesicle or a granule which is then transported along microtubules or by some other means. It is also found that each vesicle or granule typically contains several of these molecules (examples are: mRNA molecules - several estimated per granule [20,21]; cholesterol molecules which are transported in low-density lipoproteins [2] - approximately 1500 per lipoprotein).

Generally an active means of transport is not exclusively linked with the transport of large substrate molecules. The cell being a highly compartmentalized and dynamic entity requires for its survival the precise transport of certain molecules from one compartment to another and a regulation of this transport depending on its current physiological state. Brownian motion leads to an isotropic movement of molecules down the concentration gradient and to a consequent damping of the substrate concentration with distance. In contrast active transport provides a directed (anisotropic) means of transport with little or no loss of substrate with distance, is independent of the concentration gradient and it is also easily amenable to modulation.

Hence it follows that a more general process modeling molecular entry into an intracellular compartment is one in which *M *molecules are fed into the compartment at a rate ; the latter rate constant is the rate at which vesicles or granules arrive to the site of the compartment (Figure 1(B)). The total mean substrate input rate is then *k*_*in *_= *M*. The special case *M *= 1 corresponds to Model I. We construct the relevant master equation and employ the system-size expansion as for the previous model (see Methods for details); it is found that the deterministic rate equations are exactly Eqs. (1)-(4) i.e. at the macroscopic level, given two reactions occurring in two different compartments, both with the same total mean substrate input rate *k*_*in *_but one occurring via diffusion (e.g. *M *= 1,  = 1) and the other via active transport (e.g. *M *= 10,  = 0.1), cannot be distinguished. However if the compartment volumes become small, then once again we find corrections to the MM equation and interestingly these corrections are sensitive to the mode of transport. The relationship between the normalized reaction velocity predicted by the MM equation (*α*_*M*_) and the actual normalized reaction velocity (*α*), as predicted by our theory, is given by Eq. (7) together with:(9)

This suggests that generally deviations from the predictions of the MM equation increase with the carrying capacity, *M*, of the vesicle or granule. To compare the effects of active transport and diffusion on the kinetics, we set *M *= 50 and adjusted  so that in all cases, the total mean substrate input rate for model II is equal to *k*_*in*_, the input rate of Model I (i.e. the two models would be indistinguishable from a macroscopic point of view). Using the same procedure as for Model I, we computed the maximum percentage error using Eqs. (7) and (9) and also from simulations. The results are summarized in Table 2. Notice that now the deviations from the MM equation are much larger than before, running into hundreds of percent rather than the tens as for Model I. Because of the increase in substrate fluctuations, the quantitative accuracy of the theory is now less than before; it generally fares very well for compartments with diameters larger than ~100 nm and *K*_*M *_larger than ~100 *μM*. Nevertheless in all cases theory does correctly predict a large increase in discrepancy between the reaction velocities given by the deterministic MM equation and those from stochastic simulation compared to the case of Model I. The intuitive reason behind these increases in discrepancy is that substrate which is input in bursts enhances correlations between successive enzyme-substrate events.

**Table 2:** Maximum Percentage error in reaction velocity from prediction of the MM equation for Model II.

D/nm	*K*_*M *_= 10 *μM*	100 *μM*	1000 *μM*	Copy No.
50	*225.40*	*152.83 ***[291.56]**	*45.43*	10
100	*161.59 ***[331.66]**	*52.74 ***[58.39]**	*6.82 ***[6.99]**	10
200	*65.09*	*8.45 ***[8.50]**	*0.88*	10

50	*32.97*	*30.17 ***[61.66]**	*18.14*	100
100	*30.78 ***[66.03]**	*19.76 ***[24.52]**	*5.57 ***[6.06]**	100
200	*21.27*	*6.61 ***[6.91]**	*0.85*	100

The copy number indicates the total number of enzyme molecules per compartment. Values in bold and in square brackets are those estimated by simulation; the italic values are obtained from the theoretical expressions, Eqs. (7) and (9).

The explicit dependence of the reaction velocity on substrate concentration is complex and generally requires the solution of the cubic polynomial encapsulated by Eqs. (7) and (9). However for small substrate concentrations, the equations simplify to a simple linear equation:(10)

**Figure 3:**
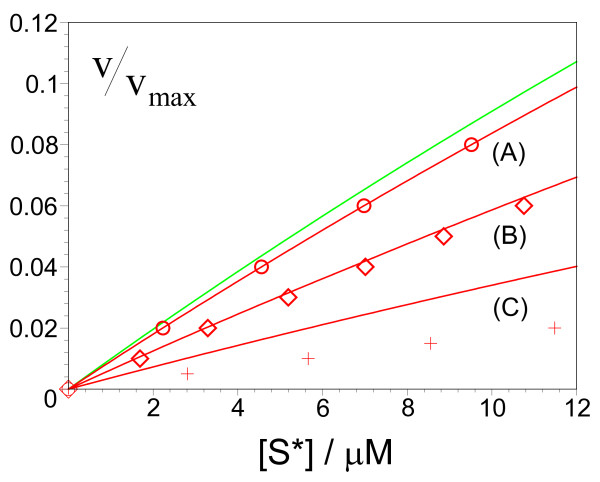
**Deviations from the predictions of the MM equation for vesicle-mediated substrate transport**. (Model II) Testing the validity of the MM relationship at small substrate concentrations for the case in which substrate input into compartments occurs in bursts. The data is for 10 enzymes with *K*_*M *_= 100 *μM *in compartments of diameter (A) 200 nm (circles), (B) 100 nm (diamonds) and (C) 50 nm (crosses); substrate is input *M *= 50 molecules at a time. The deterministic prediction for all three cases is the same MM equation shown by the green curve. In contrast, the stochastic models, [Eqs. (7) and(9)], predict different rate equations for each case (red solid lines). Data points are obtained by stochastic simulation (see Methods for details). Note that *v*/*v*_*max *_= *α*_*M *_and α for solid green and red lines respectively.

Note that if the MM equation was correct, one would expect *α *= [S*]/*K*_*M*_. Indeed Eq. (10) reduces to the latter prediction in the limit of large volumes. Note also that this renormalization of the proportionality constant occurs only if the substrate input occurs in bursts, i.e. *M *> 1. These predictions of our theory are verified by simulations (Figure 3).

### Model III: Michaelis-Menten reaction with competitive inhibitor occurring in a compartment volume of sub-micron dimensions. Substrate input as in previous models

In this last section, we further build on the previous two models by adding competitive inhibitors to the intracellular compartment in which enzymes are localized. A competitive inhibitor, *I*, is one which binds reversibly to the active site of the enzyme (forming a complex *EI*), thus preventing substrate molecules from binding to the enzyme and slowing down catalysis (Figure 1(C)). In standard textbooks and in the literature, this is typically modeled by the set of reactions (see for example [22]): , where *k*_4 _=  [*I*] and [*I*] is the inhibitor concentration. Note that it is implicitly assumed that inhibitor is in such abundance that the second-order bimolecular reaction between inhibitor and enzyme can be replaced by a pseudo first-order reaction with constant inhibitor concentration. We shall assume the same in our model. Substrate input into the compartment is considered to occur as in Model II since this encapsulates that of Model I as well. The deterministic model of this set of reactions leads to a MM equation of the form:(11)

where *β *= [*I*]/*K*_*i *_and *K*_*i *_= *k*_3_/ is the dissociation constant of the inhibitor. The perturbative solution of the master equation describing the system is now significantly more involved than in previous models; the underlying reason for this is that the computation of the noise correlators to order Ω^0 ^requires the inversion of a 6 × 6 matrix as opposed to a 3 × 3 one in previous models (see Methods for details). The analysis predicts corrections to the MM equation by postulating a new mesoscopic rate equation having the form of Eq. (7) together with:(12)

where *c*_*i *_and *d*_*i *_are coefficients with a complex dependence on the various enzyme parameters (these are given in full in the Methods Section). Table 3 shows the maximum percentage error computed using Eqs. (7) and (12) and also from simulations for the cases in which substrate input occur a molecule at a time and in bursts of 50 at a time. The parameter values chosen in the simulations and calculations (see caption of Table 3) are typical for many enzymatic processes: the bimolecular rate coefficients span the range 10^6 ^- 10^9^*s*^-1^*M*^-1^[22], the backward decay processes are in the middle of the range 10-10^5^*s*^-1 ^[22], the inhibitor concentration is ten times larger than the total enzyme concentration (satisfying the implicit assumption that the inhibitor is in significantly larger concentration than free enzyme), and the intracompartmental enzyme concentrations are in the range 4 - 255 *μM*. The deviations from the MM equation in this case are more severe than in the previous two models, this being due to non-zero correlations between substrate and the complex *EI *in addition to the already present correlations between substrate and complex *C*. Note that the agreement between theory and simulations is overall better than in previous models, even when the burst size is large, *M *= 50. As mentioned in the section for Model I, discrepancies between theory and simulation are generally found to decrease with increasing *K*_*M*_; for the case of competitive inhibition, the effective *K*_*M *_of the reaction is significantly larger than that of the enzyme (see Eq. (11)), which explains the increased agreement between theory and simulations for Model III compared to the previous two models.

**Table 3:** Maximum Percentage error in reaction velocity from prediction of the MM equation for Model III.

D/nm	*K*_*M *_= 10 *μM*	100 *μM*	1000 *μM*	M (burst size)
50	*67.8 ***[76.8]**	*67.8 ***[76.5]**	*67.8*	1
100	*20.8 ***[26.4]**	*20.6 ***[26.1]**	*20.6*	1
200	*2.8*	*2.7*	*2.7*	1

50	*1001.8*	*234.9 ***[169.4]**	*86*	50
100	*343.7 ***[345.5]**	*73.4 ***[75.2]**	*26.2 ***[31.5]**	50
200	*71.4*	*11.3 ***[11.5]**	*3.6*	50

The total number of enzyme molecules per compartment is ten in all cases. Values in bold and in square brackets are those estimated by simulation; the italic values are obtained from the theoretical expressions, Eqs. (7) and (12). The parameters are: k_0_ = 10^9^ s^-1^ M^-1^, k_1_ = k_3_ = 1000 s^-1^, k_4_^0^ = 10^7^ s^-1^ M^-1^, and [I] = 10 [E_T_].

**Figure 4:**
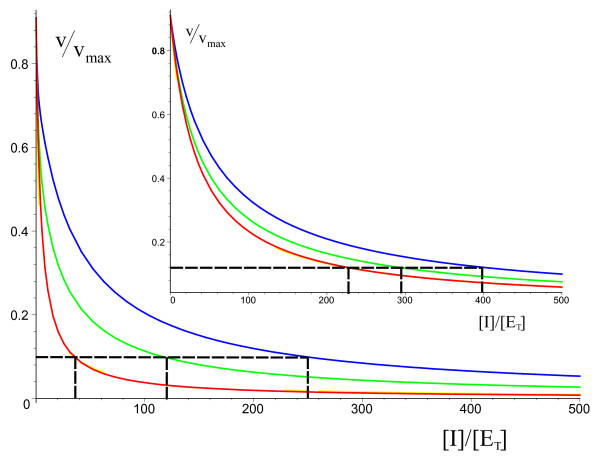
**Effects of intrinsic noise on the inhibition of enzyme activity in small compartments**. (Model III) Plots of normalized enzyme activity versus normalized inhibitor concentration (measured in units of the total enzyme concentration [*E*_*T*_]) for 10 enzymes with *K*_*M *_= 100 *μM *in compartments of diameter 50 nm and 100 nm (inset). The colors correspond to: (red) MM equation; (green) stochastic model, *M *= 20; (blue) stochastic model, *M *= 50. The latter two curves are those predicted by theory [Eqs. (7) and(12)]. Parameters same as mentioned in caption of Table 3 (except for [I], which is a variable in the present case). Substrate concentrations chosen so that at [I] = 0, *v*/*v*_*max *_= 0.909 in all cases. Black dashed lines contrast the inhibitor concentration required to decrease enzyme activity from 0.909 to 0.1 as predicted by the MM equation and the stochastic models. Note that *v*/*v*_*max *_= *α*_*M *_and *α *for solid red and blue/green lines respectively.

A significant number of drugs suppress a chain of biochemical reactions by reducing the activity of key enzymes in the pathway via competitive inhibition [17]. The conventional method to estimate the required concentrations of these inhibitors involves plotting the variation of the enzyme activity with inhibitor concentration, [*I*], using the MM equation; the substrate concentration is kept fixed and is chosen so that at [*I*] = 0, the reaction velocity is close to the maximum, *v*_*max*_. Since there are significant corrections to the MM equation when reactions occur in intracellular compartments, it is not clear how accurate are estimates of [*I*] based upon it. Figure 4 compares the enzyme activity curve based on the MM equation with the theoretical predictions for the corrected enzyme activity curves based on the mesoscopic rate equation embodied by Eqs. (7) and (12), for compartments of diameter 50 nm and 100 nm (inset) and for substrate input burst sizes of *M *= 20 and 50. The substrate concentration is chosen so that at [*I*] = 0, *v*/*v*_*max *_= 0.909 in all cases. We find that generally as the burst size increases, the actual inhibitor concentration needed to suppress enzyme activity by a given amount is larger than that estimated by the MM equation; this discrepancy decreases with increasing compartment volume. For the example in Figure 4, for the case in which substrate is input into the compartment in bursts of *M *= 50, the actual inhibitor concentration needed to decrease the enzyme activity from 0.909 to 0.1 is approximately 7 times larger than the MM estimate; if the compartment diameter is doubled (inset of Figure 4), the actual inhibitor concentration needed is less than twice that of the MM estimate. Generally we find that for the typical parameter values of enzymatic reactions, the corrections to the enzyme-activity curves can be neglected for compartments larger than about 200 nm in diameter.

## Discussion and Conclusion

In this last section we discuss some fine points regarding: (i)the assumptions behind the use of master equations which throws light on the range of use of the derived mesoscopic equations, (ii) the use of the system-size expansion to perturbatively solve the master equation and (iii) the assumption of steady-state metabolic conditions. We conclude by placing our work in the context of previous recent studies of stochastic enzyme kinetics and discuss possible experiments to verify some of the conclusions we have reached.

We have implicitly assumed throughout the article that a single (global) master equation model suffices to capture the deviations from classical kinetics due to fluctuations in chemical concentrations inside a single subcellular compartment. As noted by Baras and Mansour [23], "the global master equation selects the very limited class of exceptionally large fluctuations that appear at the level of the entire system, disregarding important nonequilibrium features originated by local fluctuations." Hence the results presented here necessarily underestimate the possible deviations from classical kinetics, in particular the local fluctuations due to diffusion of molecules inside the compartment. These local fluctuations are typically small for reaction-limited processes (as in this article) but significant for diffusion-limited ones. To capture them effectively, one would be required to spatially discretize the compartment into many small elements and describe the reaction-diffusion processes between these elements by means of a multivariate master equation [12,23]. The latter is known as a reaction-diffusion master equation; typically it does not allow detailed analytical investigation as for a global master equation and one is limited to stochastic simulation. Use of the global master equation is also restricted for compartments which are not too small: in particular the linear dimensions of the compartment should be larger than the average distance traveled by a molecule before undergoing a successful reaction with another molecule i.e. the length scale is much larger than that inherent in molecular dynamics simulation [23].

We have applied the systematic expansion due to van Kampen to perturbatively solve the master equation. It is sometimes *a priori *assumed that because this expansion is about the macroscopic concentrations, it cannot give information regarding the stochastic kinetics of few particle/small volume systems. This is true if one restricts oneself to the expansion to order Ω^0 ^i.e. the linear-noise approximation; this is commonly the case found in the literature since the algebra becomes tedious if one considers more terms. However we note that as argued and shown by van Kampen himself [12], terms beyond the linear-noise approximation in the system-size expansion add terms to the fluctuations that are of order of a single particle relative to the macroscopic quantities and are essential to understanding how fluctuations are affected by the presence of non-linear terms in the macroscopic equation (substrate-enzyme binding in our case). In our theory we went beyond the linear-noise approximation. We find that the predicted theoretical results are in reasonable agreement, in many cases (comparison of bold and italic values in Tables 1, 2 and 3), with stochastic simulations of just a few tens of enzyme molecules in sub-micron compartments, which justifies our methodology.

We have also imposed metabolic steady-state conditions inside the subcellular compartment. Technically this is convenient since in such a case one does not deal with complex transients. Also since under such conditions the MM equation is exact from a deterministic point of view, it provides a very useful reference point versus which to accurately compute deviations due to intrinsic noise. In reality one may not always have steady-state conditions inside cells, this depending strongly on the rate of substrate input relative to the maximum rate at which the enzyme can process substrate. Another possibility is that one is dealing with a batch reaction i.e. one in which a number of substrate molecules are transported at one go and just once to the subcellular compartment (e.g. via vesicle-mediated transport) and the reaction proceeds thereafter without any further substrate replenishment. This latter scenario is compatible with the presentation of the MM equation typical in standard physical chemistry textbooks. The MM equation is then an approximation (not exact as in steady-state case) to the deterministic kinetics, when substrate is present in much larger concentration than enzyme. This case is currently under investigation using the same perturbative framework used in this article.

We note that this is not the first attempt to study stochastic enzyme kinetics. The bulk of recent studies [24-27] have focused on understanding the kinetics of a Michaelis-Menten type reaction catalyzed by a single enzyme molecule. Deviations from classical kinetics were found to be most pronounced when one takes into account substrate fluctuations [26]. These pioneering studies were restricted to a single-enzyme assisted reaction which reduces complexity thereby making it ideal from a theoretical perspective; since the reaction is dependent on just a single enzyme molecule one also finds maximum deviations from deterministic kinetics. In reality, it is unlikely to find just one enzyme molecule inside a subcellular compartment - as mentioned in the introduction a physiological concentration of just a few hundred micromolar would correspond to few tens inside the typically smallest subcellular compartment. It is also the case that diffusion may not always be the main means of substrate transport to the compartment and that the reaction maybe more complex than the simple Michaelis-Menten type reaction of these previous studies. The present study fills in these gaps by using a systematic method to derive approximate and relatively simple analytic expressions for mesoscopic rate equations describing the kinetics of the general case of *N *enzyme molecules in a subcellular compartment with or without active transport of substrate and in the presence of enzyme inhibitors. Most importantly our approach shows the effects of intrinsic noise on the kinetics can be captured via effective ordinary differential equations. This enables quick estimation of the magnitude of stochastic effects on reaction kinetics and thus gives insight into whether a model or parts of a model should be designed to be stochastic or deterministic without the need for extensive stochastic simulation. In the present study, this approach enabled us to readily compute, for the first time, the deviations from deterministic kinetics for a broad range of realistic *in vivo *parameter constants (Tables 1, 2 and 3), a task which would be considerably lengthy if one had to rely solely on data obtained from ensemble-averaged stochastic simulations.

We conclude by briefly discussing possible experiments which can verify the predictions made in this article. It is arguably not an easy task to perform the required experiments in real-time in a living cell. A viable alternative would consist of monitoring reaction kinetics inside single artificially-made vesicles. Pick et al [8] have shown that the addition of cytochalasin to mammalian cells induces them to extrude from their plasma membrane minuscule vesicles of attolitre volume with fully functional cell surface receptors and also retaining cytosolic proteins in their interior. The change in the intra-vesicular calcium ion concentration in response to surface ligand binding was measured using fluorescence confocal microscopy (FCM). Since the vesicle sizes are of typical small sub-cellular compartment dimensions (1 attolitre corresponds to a spherical vesicle of approximate diameter 120 nm) and FCM allows the measurement of the concentration of a fluorescent probe (via a calibration procedure), this experimental technique appears ideal to verify the predictions of Model I and of Model III for the case of diffusive substrate transport. Model II and Model III with vesicle-transport of substrate are probably much more challenging to verify since one then needs to construct the *in vitro *equivalent of microtubules. This is within the scope of synthetic biology and may be a possibility in the next few years.

## Methods

We here provide full details of the calculations reported in the Results section. The system size-expansion which is at the heart of the analysis has to-date not been applied extensively to biological problems and thus we go into some detail in its elucidation in Sub section I, which is dedicated exclusively to Model I. For other recent applications of the general method in the context of reaction kinetics, see for example [28] and [29]. Subsections II and III (treating Model II and Model III, respectively) naturally build on the results of the first subsection and thus we only give the main steps of the calculations in these last two cases. Sub section IV has a brief discussion of the simulation methods used to verify the theoretical results.

### Model I: Michaelis-Menten reaction occurring in a compartment volume of sub-micron dimensions. Substrate input into compartment is modeled as a Poisson process

The reaction scheme is . The stochastic description of this system is encapsulated by the master equation which is a differential equation in the joint probability function *π *describing the system:(13)

where *π *= *π*(*n*_*C*_, *n*_*P*_, *n*_*S*_), *n*_*X *_is the integer number of molecules of type *X *(where *X *= *C*, *P*, *S*), Ω is the compartment volume, and  are the step operators defined by their action on a general function *g*(*n*_*X*_) as: *g*(*n*_*X*_) = *g*(*n*_*X *_± 1). Note that the relevant variables are three, not four: the integer number of molecules of free enzyme (*n*_*E*_) is not an independent variable due to the fact that the total amount of enzyme is conserved. The master equation cannot be solved exactly but it is possible to systematically approximate it by using an expansion in powers of the inverse square root of the volume of the compartments. This is generally called the system-size expansion [12].

The method proceeds as follows. The stochastic quantity, *n*_*X*_/Ω, fluctuates about the macroscopic concentrations [X]; these fluctuations are of the order of the square root of the number of particles:(14)

Note that since *n*_*E *_+ *n*_*C *_= *constant*, it follows that *n*_*E *_= Ω[*E*] - Ω^1/2^ϵ_*C*_. The joint distribution function and the operators can now be written as functions of the new variables, ϵ_*X*_, giving: *π *= Π(ϵ_*C*_, ϵ_*P*_, ϵ_*S*_, *t*) and ; using these new variables the master equation Eq. (13) takes the form:(15)

where(16)

Note that in Eq. (18) terms which involve products of first and second-order derivatives, third-order derivatives or higher have been omitted - these do not affect the low-order moment equations which we will be calculating.

#### Analysis of Ω^1/2 ^terms

The terms of order Ω^1/2 ^are the dominant ones in the limit of large volumes. By equating both terms of this order on the right and left hand sides of Eq. (15) and using Eq. (16), one gets the deterministic rate equations:(19)

These are exactly those which one would write down based on the classical approach whereby one ignores molecular discreteness and fluctuations. This is an important benchmark of the method since it shows that it gives the correct result in the limit of large volumes. On a more technical note, the cancelation of these two terms of order Ω^1/2 ^makes Eq. (15) a proper expansion in powers of Ω^-1/2^. By imposing steady-state conditions we have the Michaelis-Menten (MM) equation:(22)

where *v*_*max *_= *k*_2 _[*E*_*T*_] is the maximum reaction velocity, [*E*_*T*_] = [*E*] + [*C*] is the total enzyme concentration which is a constant at all times and *K*_*M *_= (*k*_1 _+ *k*_2_)/*k*_0 _is the Michaelis-Menten constant.

#### Analysis of Ω^0 ^terms

To this order, the master equation is a multivariate Fokker-Planck equation whose solution is Gaussian and thus fully determined by its first and second moments. The equations of motion for these moments can be straightforwardly obtained from the master equation to this order, leading to a set of coupled but solvable ordinary differential equations:(23)

where,(25)

Note that the matrices and vectors in the above equations have been reduced to a simpler form by the application of a few row operations. Note also that these equations are independent of ϵ_*p *_since the product-forming step is irreversible and hence the fluctuations in substrate and complex are necessarily decoupled from its fluctuations. At the steady-state, it is found that ⟨ϵ_*S*_, _*C*_⟩ → 0. From Eq. (14), it is clear that this implies that to this order the average number of substrate molecules per unit volume, ⟨*n*_*S*_/Ω⟩, is simply equal to the macroscopic concentration, [*S*]. The same applies for complex molecules. Hence to this order in the system-size expansion there cannot be any corrections to the macroscopic equations or to the MM equation. By writing the macroscopic concentrations in Eqs. (24) and (25) in terms of *k*_*in *_and solving, one obtains the variance and covariance of the fluctuations about the steady-state macroscopic concentrations. We here only give the result for the covariance since this will be central to our discussion later on:(26)

where *α *= *k*_*in*_/*v*_*max *_is the normalized reaction velocity of the enzyme.

#### Analysis of Ω^-1/2 ^terms

The system-size expansion is almost never carried out to this order because of the algebraic complexity typically involved, however it is crucial to find finite volume corrections to the deterministic rate equations and in particular to the MM equation. Using the master equation to this order, the first moment of the complex concentration is governed by the equation of motion:(27)

Now the production of product *P *from complex occurs through a decay process which necessarily has to be described by a linear term of the form: *k*_*in *_= *k*_2_⟨*n*_*C*_/Ω⟩ (the steady-state condition). Since the steady-state macroscopic complex concentration is equal to [*C*] = *k*_*in*_/*k*_2_, then it follows that to any order in the expansion we have ⟨ϵ_*C*_⟩ = 0. This is always found to be the case in simulations as well. Hence it immediately follows from Eq. (27) that the average of fluctuations about the macroscopic substrate concentration are non-zero and given by:(28)

From a physical point of view, this indicates that the steady-state concentration of substrate in the compartment is not equal to the value predicted by the MM equation (i.e. [S]) and hence the non-zero value of the average of the fluctuations about [S]. The real substrate concentration inside the compartment is obtained by substituting Eqs. (28) and (26) in Equation (14), leading to:(29)

#### An alternative mesoscopic rate equation replacing the MM equation

The renormalization of the steady-state substrate concentration indicates the breakdown of the MM equation; this phenomenon occurs because of non-zero correlations between noise in the substrate and enzyme concentrations, ⟨ϵ_*S*_ϵ_*C*_⟩, which the MM equation implicity neglects. To obtain the alternative to the latter, one needs to obtain a relationship between the normalized reaction velocity, *α *and the real substrate concentration ⟨*n*_*S*_/Ω⟩; writing [*S*] in terms of α and substituting in Eq. (29), one obtains this new relation:(30)

Note that in the limit of large volumes, the second term on the left hand side of Eq. (30) becomes vanishingly small and we are left with the MM equation. In the results section the quantity on the right hand side of Eq. (30) is referred to as *α*_*M *_since this is the normalized reaction velocity which would be predicted by the MM equation given the measured substrate concentration ⟨*n*_*S*_/Ω⟩ inside the compartment. A quick estimate of the magnitude of the error that one is bound to incur by using the conventional MM equation can be obtained by substituting *α *= 1/2 (i.e. enzyme is half saturated with substrate) in Eqs. (30) and (31), solving for *α*_*M *_and then using this value to compute the fractional error e = 1 - *α*_*M*_/*α*. This leads to the simple expression:(32)

We finish this section by noting that Eq. (30) will be found to be valid generally and not only for the simple Michaelis-Menten scheme treated in this section; the details of the reaction network come in through the form of Eq. (31) which is reaction-specific.

### Model II: Michaelis-Menten reaction occurring in a compartment volume of sub-micron dimensions. Substrate is input into compartment in groups or bursts of *M *molecules at a time

A natural generalization of Model I which has direct biological application is when substrate molecules are fed into the compartment *M *at a time with mean rate . The total mean substrate input rate is then equal to *k*_*in *_= *M*. The master equation for this process is Eq. (13) with the first term on the right hand side replaced by . This leads to the following change in the expression for *a*_2 _(Eq. 17):(33)

Note that since the expression for *a*_1 _(Eq. 16) is unchanged, the deterministic equations are precisely the same as those of Model I. However now the fluctuations about the macroscopic substrate concentration are enhanced by a factor *M*; consequently the entries in the vector B in Eq. (25) need the change *k*_*in *_→ *k*_*in*_*M*. The analysis proceeds in the same manner as before. The mesoscopic rate equation replacing the MM equation is now given by Eq. (30) together with:(34)

The fractional error rate evaluated at *α *= 1/2 gives:(35)

This clearly shows that generally larger deviations from the predictions of the MM equation are expected in this case compared to those computed for Model I.

### Model III: Michaelis-Menten reaction with competitive inhibitor occurring in a compartment volume of sub-micron dimensions. Substrate input as in two previous models

Competitive inhibition is modeled by the set of reactions: , where *k*_4 _= [*I*] and [*I*] is the inhibitor concentration (similar models have been studied by Roussel and collaborators [30,31] in the context of biochemical oscillators though these assume *M *= 1). In the rest of this section, we change the notation of enzyme-inhibitor complex from *EI *to *V*, just to make the math notation easier to read. The substrate input into the compartment is considered to occur as in Model II since this encapsulates that of Model I as well. The master equation for this system is:(36)

The change of variables from *n*_*X *_to ϵ_*X *_is done as before, however note that now the conservation law for enzyme is different than in the two previous models. The total enzyme concentration is now equal to [*E*_*T*_] = [*E*] + [*C*] + [*V*] from which it follows that *n*_*E *_= Ω[*E*] - Ω^1/2^(ϵ_*C *_+ ϵ_*V*_). The description is chosen to be in terms of numbers of molecules of types *C*, *S *and *V *and thus *E *being a dependent variable does not show up explicitly in the step operators of the master equation above.

Due to the significant number of changes in the terms of the expansion from those of previous models, we will show the equivalent of Eqs. (15)-(18) in full. The master equation in the new variables ϵ_*X *_is given by:(37)

where(38)

#### Analysis of Ω^1/2 ^terms

As for previous models, these terms give the macroscopic equations. Equating both terms of this order on the right and left hand sides of Eq. (37) and using Eq. (38), one obtains:(41)

In the steady-state we have the Michaelis-Menten (MM) equation:(45)

where *β *= [*I*]/*K*_*i *_and *K*_*i *_= *k*_3_/ is the dissociation constant of the inhibitor.

#### Analysis of Ω^0 ^and Ω^-1/2 ^terms

The equations for the first moments are easily obtained and we shall not reproduce them here; suffice to say that at steady-state, it is found that ⟨ϵ_*S*_, *C*, *V*⟩ → 0 which implies that to this order in the system-size expansion there cannot be any corrections to the macroscopic equations or to the MM equation. The addition of a new species, *V*, does however substantially increase the algebraic complexity in the equations of motion for the second moments computed using terms up to order Ω^0^. In particular the matrix A is now a 6 × 6 matrix, rather than the 3 × 3 matrix of the previous two models.(46)

where,(47)

and(48)

In the above equations we have defined *k' *= *k*_3 _+ *k*_4_. Note also that the system of equations has been simplified through the application of a few row operations.

Now to next order, i.e. Ω^-1/2^, the first moments of the concentrations of molecules of type *C *and *V *are governed by the equation of motions:(49)

As in previous models, since the production of product *P *from complex occurs through a decay process, it follows that at steady-state, ⟨ϵ_*C*_⟩ = 0 which also implies ⟨ϵ_*V*_⟩ = 0 from Eq. (50). Hence it follows from Eq. (49) that ⟨ϵ_*S*_⟩ = [⟨ϵ_*S*_ϵ_*C*_⟩ + ⟨ϵ_*S*_ϵ_*V*_⟩]/Ω^1/2 ^[*E*]. The two cross correlators can be estimated to order Ω^0 ^by solving Eqs. (46)-(48). The non-zero value of ⟨ϵ_*S*_⟩ implies a renormalization of the substrate concentration inside the compartment and hence to a new rate equation replacing the MM equation. This is obtained exactly in the same manner as previously shown for Model I. The mesoscopic rate equation is found to be given by Eq. (30) together with:(51)

where the numerator coefficients are given by:(52)

and the denominator coefficients by:(57)

Note that  = 0 such that at *α *= 0, there is no correction to the MM equation i.e. *α*_*M *_= 0 also. The case *β *= 0 reduces to Model II, i.e. *f*(*α*) is given by Eq. (34).

#### Stochastic simulation

In this section we briefly describe the simulation methods used to verify the theoretical results which are described in detail in the Results section. All simulations were carried out using Gillespie's exact stochastic simulation algorithm, conveniently implemented in the standard simulation platform, Dizzy [32].

The data points in Figure 2 were generated by iterating the following four-step procedure: (i) pick a value for *α *between 0 and 1. This gives the substrate input rate *k*_*in *_= *αv*_*max*_; (ii) run the simulation and measure the ensemble-averaged substrate concentration, ⟨*n*_*S*_/Ω⟩ = [S*] at long times; (iii) compute *α*_*M *_using the MM equation, *α*_*M *_= [S*]/([S*]+ *K*_*M*_); (iv) compute the absolute percentage error *R*_*p *_= 100|(1 - *α*_*M*_/*α*)|. The solid curves in Figure 2 were obtained by numerically solving the cubic polynomial in *α *given by Eqs. (7) and (8) in the Results section for given values of *α*_*M *_and then using the above expression for *R*_*p*_. Figure 3 is generated in the same manner as Figure 2, except that: in step (i) we fix *M *and pick a value for *α *between 0 and 1. Since *k*_*in *_= *M*, the required simulation parameter is  = *αv*_*max*_/*M*; step (iv) is not needed. The solid curves were obtained by numerically solving the cubic polynomial in *α *given by Eqs. (7) and (9) in the Results section for given values of [S*]. The y-axis for this figure is *v*/*v*_*max *_= *α*_*M *_for the MM equation and *v*/*v*_*max *_= *α *for the stochastic model. Figure 4 is obtained by numerically solving the quintic polynomial in *α *given by Eqs. (7) and (12) in the Results section together with the coefficients given by Eqs. (52)-(62) in the present section; the inhibitor concentration, [*I*], is varied while the substrate concentration, [S*], is kept fixed. The substrate concentration is chosen so that at [*I*] = 0, *v*/*v*_*max *_= 0.909 in all cases. Note that for models I and II, *α*_*M *_= [S*]/([S*] + *K*_*M*_) while for Model III, *α*_*M *_= [S*]/([S*] + (1 + *β*)*K*_*M*_). Note that the error bars are very small on the scale of the figures and thus are not shown.
